# Comparative Thermal Research on Energetic Molecular Perovskite Structures

**DOI:** 10.3390/molecules27030805

**Published:** 2022-01-26

**Authors:** Jing Zhou, Junlin Zhang, Shaoli Chen, Fengqi Zhao, Lili Qiu, Zihui Meng, Li Ding, Bozhou Wang, Qing Pan

**Affiliations:** 1 Xi’an Modern Chemistry Research Institute, Xi’an 710065, China; zhoujing19872006@163.com (J.Z.); asierchen@163.com (S.C.); zhaofqi@163.com (F.Z.); dingli403@sina.com (L.D.); IR204@tom.com (Q.P.); 2 School of Chemistry and Chemical Engineering, Beijing Institute of Technology, Beijing 102488, China; mengzh@bit.edu.cn

**Keywords:** confined effect, decomposition mechanisms, DSC-TG-FTIR-MS quadruple technology, molecular perovskites, thermal research

## Abstract

Molecular perovskites are promising practicable energetic materials with easy access and outstanding performances. Herein, we reported the first comparative thermal research on energetic molecular perovskite structures of (C_6_H_14_N_2_)[NH_4_(ClO_4_)_3_], (C_6_H_14_N_2_)[Na(ClO_4_)_3_], and (C_6_H_14_ON_2_)[NH_4_(ClO_4_)_3_] through both calculation and experimental methods with different heating rates such as 2, 5, 10, and 20 °C/min. The peak temperature of thermal decompositions of (C_6_H_14_ON_2_)[NH_4_(ClO_4_)_3_] and (C_6_H_14_N_2_) [Na(ClO_4_)_3_] were 384 and 354 °C at the heating rate of 10 °C/min, which are lower than that of (C_6_H_14_N_2_)[NH_4_(ClO_4_)_3_] (401 °C). The choice of organic component with larger molecular volume, as well as the replacement of ammonium cation by alkali cation weakened the cubic cage skeletons; meanwhile, corresponding kinetic parameters were calculated with thermokinetics software. The synergistic catalysis thermal decomposition mechanisms of the molecular perovskites were also investigated based on condensed-phase thermolysis/Fourier-transform infrared spectroscopy method and DSC-TG-FTIR-MS quadruple technology at different temperatures.

## 1. Introduction

Energetic materials, which release high amount of the stored chemical energy and structurally combine both fuel and oxidizer parts, have contributed enormously to the progress of military and civil industry [1,2,3]. Most conventional energetic materials normally consist of a carbon core (fuel moiety) incorporating covalently bonded oxidizer groups (oxidizer moiety) such as nitro, nitramine, nitrate ester etc. On explosion, an internal redox reaction occurs between these oxidative groups and the carbon cores, forming gaseous products and generating large amount of heat [4]. The past decades witnessed a fast growth of advanced energetic materials [5,6]; however, the complicated syntheses, expensive costs, as well as the lack of systemic and in-depth property studies, have seriously hindered their wide applications.

**Figure 1:**
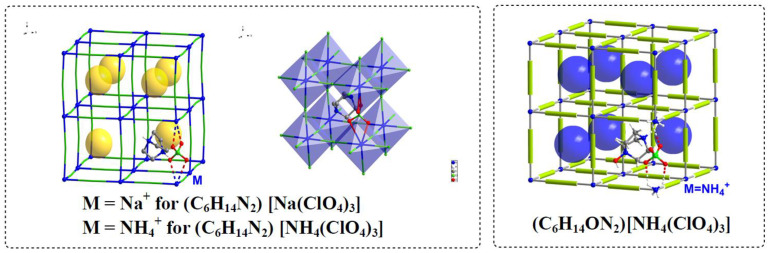
Structures of the energetic molecular perovskites.

Molecular perovskites have been demonstrated as promising high explosives and solid propellants, which rely on the self-assembly of diverse molecular components into specified ternary crystal structures and exhibit some unique advantages like easy scale-up preparations at a low cost, increased pack efficiency (and crystal density), and optimized oxygen balance [7,8,9,10,11]. Comparative thermal research on the thermal properties and thermal behaviors are crucial to the practical applications of energetic materials [12,13]. Our previous thermal research of (C_6_H_14_N_2_)[NH_4_(ClO_4_)_3_] has indicated a confined effect of the protonated DABCO cation being locked in the cage skeleton constructed by NH_4_^+^ and ClO_4_^−^ ions that renders (C_6_H_14_N_2_)[NH_4_(ClO_4_)_3_] an outstanding thermal stability [14]. More recently, a series of different cations, including the organic cations (the one locked in the cage, A-site cation) and inorganic cations (the one used for the construction of the cage, B-site cation), have also been beautifully introduced for the synthesis of molecular perovskites [11,15]. Obviously, the confined effect from the cage skeleton is distinctive and will be practical in the development of advanced energetic structures, therefore, we are highly interested in how the organic and inorganic cations applied will affect this effect. Herein, we reported a comparative thermal research on two chosen molecular perovskite energetic structures, (C_6_H_14_ON_2_)[NH_4_(ClO_4_)_3_] and (C_6_H_14_N_2_) [Na(ClO_4_)_3_], in which different cations were embedded (Figure 1). The thermal behaviors, the non-isothermal decomposition reaction kinetics, as well as the gaseous and solid decomposition products of (C_6_H_14_ON_2_)[NH_4_(ClO_4_)_3_] and (C_6_H_14_N_2_) [Na(ClO_4_)_3_] were investigated and compared with those of (C_6_H_14_N_2_)[NH_4_(ClO_4_)_3_] through the combination of differential scanning calorimetry (DSC), simultaneous thermal analysis (STA), solid phase in situ FTIR spectroscopy, and DSC-TG-FTIR-MS technologies [16]. Possible decomposition mechanism was also discussed based on the experiment and calculation results.

## 2. Results and Discussion

**Figure 2:**
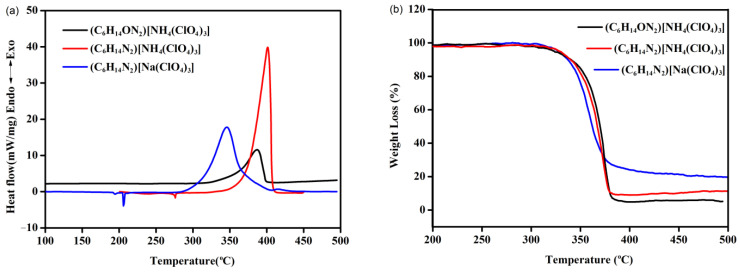
Comparative DSC (**a**) and TG (**b**) experiments on the energetic molecular perovskite structures.

Studies on thermal behaviors are the basis for obtaining practicable thermal properties of energetic materials. In our thermal research on energetic molecular perovskite structures, their thermal decomposition behaviors were investigated and compared first. As an oxidized organic cation locked in the cage skeleton of (C_6_H_14_ON_2_)[NH_4_(ClO_4_)_3_], H-OH-DABCO^2+^ (1-hydroxy-1,4-diazabicyclo[2.2.2]-octane-1,4-diium) has larger molecular volume than that of H_2_DABCO^2+^ (1,4-diazabicyclo[2.2.2]-octane-1,4-diium), which is locked in the cage skeletons of both (C_6_H_14_N_2_)[NH_4_(ClO_4_)_3_] and (C_6_H_14_N_2_)[Na(ClO_4_)_3_]. Based on the crystal structure data, the sizes of the cubic cage skeletons constructed by the inorganic cations and anions in (C_6_H_14_N_2_)[NH_4_(ClO_4_)_3_], (C_6_H_14_ON_2_)[NH_4_(ClO_4_)_3_] and (C_6_H_14_N_2_)[Na(ClO_4_)_3_] are quite close, indicating the change of the B-site cations will not change the size of the cubic cage skeleton much [17,18]. In our previous work, we assumed that the rotation of H_2_DABCO^2+^ in the cubic cage skeleton could be possible to trigger the change of crystalline form during the heating process and resulted in the weak endothermic peak in DSC curve of (C_6_H_14_N_2_)[NH_4_(ClO_4_)_3_]. A highly similar endothermic process was observed in the DSC curve of (C_6_H_14_N_2_)[Na(ClO_4_)_3_], and more interestingly, no similar endothermic process was observed in the DSC curve of (C_6_H_14_ON_2_)[NH_4_(ClO_4_)_3_] (Figure 2a). Similar endothermic peaks in DSC curves were also proved in explorations of two molecular perovskites with H_2_DABCO^2+^ as the organic cation [19,20]. Clearly, the endothermic process has close relationship with A-site cations. Seen from the crystal structure, the larger molecular volume of H-OH-DABCO^2+^ fully filled the cubic cage skeleton and, therefore, blocked the rotation of the organic cation. The peak temperature of thermal decompositions of (C_6_H_14_ON_2_)[NH_4_(ClO_4_)_3_] and (C_6_H_14_N_2_)[Na(ClO_4_)_3_] were 384 and 354 °C at the heating rate of 10 °C/min, which are lower than that of (C_6_H_14_N_2_)[NH_4_(ClO_4_)_3_] (401 °C). Nonetheless, the thermal stability of these molecular perovskite structures exceeds that of most energetic materials. Moreover, 4233 and 3457 J/g heat were released during the thermal decomposition of (C_6_H_14_ON_2_)[NH_4_(ClO_4_)_3_] and (C_6_H_14_N_2_)[Na(ClO_4_)_3_], which were lower than that of (C_6_H_14_N_2_)[NH_4_(ClO_4_)_3_] (5026 J/g), but higher than that of the most powerful high explosive molecules like HMX (1987 J/g) and CL-20 (3101 J/g) [21]. Most traditional energetic materials combine oxidative functional groups with molecular backbones (fuel moieties) through covalent bonds. In contrast, molecular perovskite structure is bound by Coulomb force between different ions. The higher heat release during the thermal decomposition of molecular perovskites indicates that the interaction of its oxidizing components with its fuel components is highly efficient. Compared with (C_6_H_14_ON_2_)[NH_4_(ClO_4_)_3_] and (C_6_H_14_N_2_)[Na(ClO_4_)_3_], (C_6_H_14_N_2_)[NH_4_(ClO_4_)_3_] exhibited a more concentrated exothermic peak shape. TG studies on (C_6_H_14_ON_2_)[NH_4_(ClO_4_)_3_] and (C_6_H_14_N_2_)[Na(ClO_4_)_3_] indicated that both organic cation and inorganic cation have significant influence on the extent of sample weight-loss. Compared with (C_6_H_14_N_2_)[NH_4_(ClO_4_)_3_], (C_6_H_14_ON_2_)[NH_4_(ClO_4_)_3_] exhibited a higher degree of weight-loss due to the oxidized organic cation leading to deeper oxidation. In contrast, (C_6_H_14_N_2_)[Na(ClO_4_)_3_] exhibited a lower degree of weight-loss than that of (C_6_H_14_N_2_)[NH_4_(ClO_4_)_3_] due to the residual non-volatile sodium salt (Figure 2b). The difference between the degree of oxidation and the remaining residue also partly explain the order of detonation performances of (C_6_H_14_ON_2_)[NH_4_(ClO_4_)_3_] > (C_6_H_14_N_2_)[NH_4_(ClO_4_)_3_] > (C_6_H_14_N_2_)[Na(ClO_4_)_3_].

**Figure 3:**
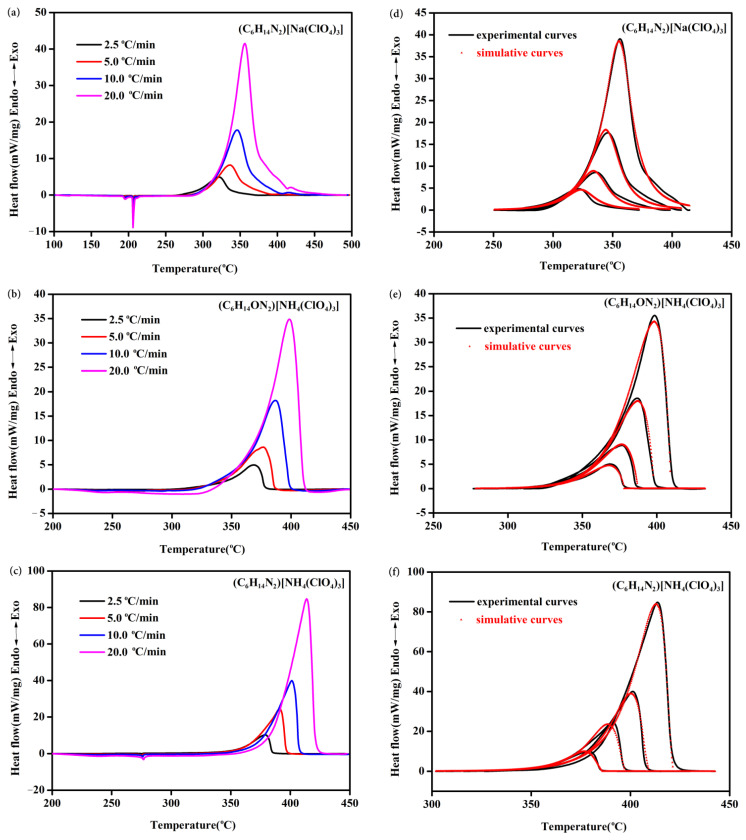
DSC experiments under different heating rates of (C_6_H_14_N_2_)[Na(ClO_4_)_3_] (**a**), (C_6_H_14_ON_2_)[NH_4_(ClO_4_)_3_] (**b**) and (C_6_H_14_N_2_)[NH_4_(ClO_4_)_3_] (**c**); The overlay of fitted and measured curves of (C_6_H_14_N_2_)[Na(ClO_4_)_3_] (**d**), (C_6_H_14_ON_2_)[NH_4_(ClO_4_)_3_] (**e**) and (C_6_H_14_N_2_)[NH_4_(ClO_4_)_3_] (**f**).

The studies of kinetic parameters play significant role in the research of thermal properties of energetic materials [22]. Non-isothermal kinetics of the thermal decompositions of (C_6_H_14_ON_2_)[NH_4_(ClO_4_)_3_] and (C_6_H_14_N_2_)[Na(ClO_4_)_3_] were investigated through DSC experiments under different heating rates of 2, 5, 10, and 20 °C/min with their thermal decomposition peaks compared with those of (C_6_H_14_N_2_)[NH_4_(ClO_4_)_3_] as shown in Figure 3a–c. Similar to many energetic materials’ thermal decomposition behaviors under different heating rates [23,24,25], the decomposition peaks of the three molecular perovskites shifted toward high temperatures with the increase of heating rate, meanwhile, both the peak shape and heat release were very close under different heating rates, indicating that the thermal decompositions of (C_6_H_14_ON_2_)[NH_4_(ClO_4_)_3_], (C_6_H_14_N_2_)[Na(ClO_4_)_3_] and (C_6_H_14_N_2_)[NH_4_(ClO_4_)_3_] were probably one-step reactions. Kinetic parameters and mechanism functions of these thermal decomposition reactions were then calculated with NETZSCH Thermokinetics Software [26]. A model-free algorithm of Friedman method was applied to predict the decomposition reaction types of (C_6_H_14_ON_2_)[NH_4_(ClO_4_)_3_], (C_6_H_14_N_2_)[Na(ClO_4_)_3_] and (C_6_H_14_N_2_)[NH_4_(ClO_4_)_3_]. Based on the preliminary calculated apparent activation energies and pre-exponential constants, various reaction models were further applied for identification of the reaction mechanisms. Multiple reaction models were tested and proved that the decomposition reactions of (C_6_H_14_ON_2_)[NH_4_(ClO_4_)_3_], (C_6_H_14_N_2_)[Na(ClO_4_)_3_] and (C_6_H_14_N_2_)[NH_4_(ClO_4_)_3_] agreed with the nth order model (Fn), Kamal-Sourour autocatalytic model (KS), and nth order autocatalytic model (Cn), respectively. Figure 3d–f showed the overlay of fitted and measured curves. The results proved that prediction curves of the three molecular perovskites coincided with their measurement results well, indicating the selected kinetic models are accurate and can be used to predict the decomposition process under designed experimental conditions. Table 1 summarizes the calculated kinetic parameters based on the selected kinetic models. According to the data in Table 1, the activation energy of (C_6_H_14_N_2_)[Na(ClO_4_)_3_] (173.9 kJ/mol) is much lower than that of (C_6_H_14_N_2_)[NH_4_(ClO_4_)_3_] (207.5 kJ/mol) and (C_6_H_14_ON_2_)[NH_4_(ClO_4_)_3_] (221.7 kJ/mol), which are quite close. The low activation energy means (C_6_H_14_N_2_)[Na(ClO_4_)_3_] is more prone to thermal decomposition under heating conditions [27]. Meanwhile, the close activation energy of (C_6_H_14_N_2_)[NH_4_(ClO_4_)_3_] and (C_6_H_14_ON_2_)[NH_4_(ClO_4_)_3_] indicates similar onset decomposition temperatures. These are basically consistent with the experimental results.

The energetic molecular perovskites of (C_6_H_14_ON_2_)[NH_4_(ClO_4_)_3_], (C_6_H_14_N_2_)[Na(ClO_4_)_3_] and (C_6_H_14_N_2_)[NH_4_(ClO_4_)_3_] structurally consist of a cubic prototype structure with a general formula of ABX_3_, in which the A-site cation is larger than the B-site one, and each B-site cation of the six is coordinated by X-site anions to form BX_6_ octahedra, which are further corner-shared to form a three-dimensional structure. The cage skeleton of the energetic molecular perovskite was constructed by the combination of inorganic cation and inorganic oxidizer anion, with the organic cation confined in the cage, presenting a new type of energetic structure that is completely different from the traditional energetic materials. Including our previous work, some of the studies mentioned above [8,9,10,14,20] have attempted to explore the thermal decomposition mechanism of individual energetic molecular perovskite based on different carefully designed computational or experimental strategies. But so far, there has been no systematic studies based on multiple molecular perovskite systems to elucidate their thermal decomposition mechanism.

**Figure 4:**
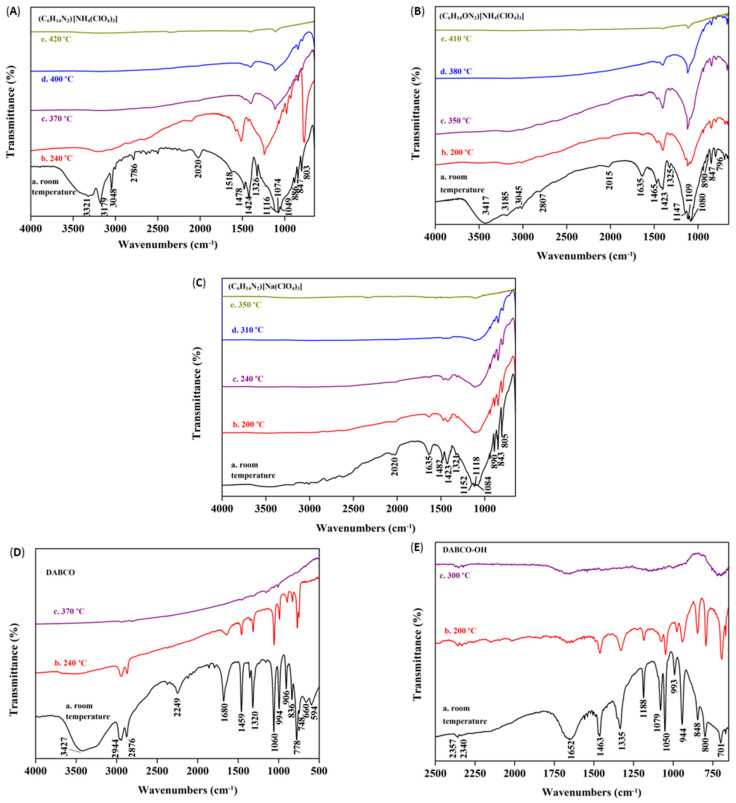
In situ FTIR spectroscopy experiments of (C_6_H_14_N_2_)[NH_4_(ClO_4_)_3_] (**A**), (C_6_H_14_ON_2_)[NH_4_(ClO_4_)_3_] (**B**), (C_6_H_14_N_2_)[Na(ClO_4_)_3_] (**C**), DABCO (**D**) and DABCO-OH (**E**) at different temperatures.

In order to systematically compare the influence of various ions on the thermal stability of molecular perovskite structures, we further studied the thermal decomposition mechanisms of (C_6_H_14_ON_2_)[NH_4_(ClO_4_)_3_] and (C_6_H_14_N_2_)[Na(ClO_4_)_3_] based on condensed-phase thermolysis/Fourier-transform infrared (in-situ FTIR) spectroscopy method at different temperatures, and compared with that of (C_6_H_14_N_2_)[NH_4_(ClO_4_)_3_]. Figure 4A–C shows the FTIR spectroscopy of the three molecular perovskites under different heating temperatures. For (C_6_H_14_ON_2_)[NH_4_(ClO_4_)_3_] and (C_6_H_14_N_2_)[NH_4_(ClO_4_)_3_], their cage skeletons was constructed by NH_4_^+^ and ClO_4_^−^, in contrast, the cage skeleton of (C_6_H_14_N_2_)[Na(ClO_4_)_3_] was constructed by Na^+^ and ClO_4_^−^. From their FTIR spectroscopy at room temperature, the broad strong signal around 1080 cm^−1^ belonged to ClO_4_^−^ in the cage skeletons constructed by NH_4_^+^-ClO_4_^−^ and Na^+^-ClO_4_^−^, respectively. It is noteworthy that the cage skeletons are rather stable and decomposed much later than the locked organic components during heating conditions. With the increase in the heating temperature, the strong signal of ClO_4_^−^ in (C_6_H_14_N_2_)[Na(ClO_4_)_3_] weakened first (around 310 °C), followed by the signal of ClO_4_^−^ in (C_6_H_14_ON_2_)[NH_4_(ClO_4_)_3_] (around 350 °C) and (C_6_H_14_N_2_)[NH_4_(ClO_4_)_3_] (around 370 °C). The order of the signal intensity attenuation of ClO_4_^−^ is opposite to the order of the stability of the cage skeleton of molecular perovskites. The strong signals of the organic cations, H-OH-DABCO^2+^ and H_2_DABCO^2+^ locked in the cage skeletons, are located around 1100 cm^−1^ (as multiple peaks overlapped with the signal of ClO_4_^−^) and around 1420 cm^−1^, 1470 cm^−1^ (two peaks). Figure 4D,E shows the FTIR spectroscopy of HO-DABCO and DABCO under different heating conditions. Clearly, DABCO disappeared fast when the heating temperature is above 240 °C through volatilization process and nothing left when heated to 370 °C, meanwhile, HO-DABCO suffered rapid decomposition at 200 °C and decomposed completely when further heated to 300 °C. But when confined in the cage [28] constructed by the inorganic components after protonated, the organic cations exhibited great stability. Although the signals of the H-OH-DABCO^2+^ and H_2_DABCO^2+^ decayed earlier than that of the ClO_4_^−^, the activated organic components still could not escape from the three-dimensional cage skeletons due to the strong Coulomb forces. With the temperature high enough, the signal of ClO_4_^−^ decayed fast, showing the cage skeletons are completely activated and fragmented, and the confined effect is greatly weakened and eliminated. At these temperatures, the organic components interacted with the ClO_4_^−^ anions and led to massive heat release which could be observed as DSC exotherm increased rapidly. Based on the comparison of the obtained experimental results, some conclusions can be drawn: (a) strength and thermal stability of the cage skeleton constructed by NH_4_^+^-ClO_4_^−^ are better than those of the cage skeleton constructed by Na^+^-ClO_4_^−^; (b) thermal stability of the organic cation H_2_DABCO^2+^ is better than that of H-OH-DABCO^2+^ locked in similar same cage skeleton; (c) for the same cage skeleton constructed by NH_4_^+^-ClO_4_^−^, the larger size of the locked H-OH-DABCO^2+^ weakened the stability of the cage skeleton, which may due to the larger size of the cation contained which can easily crack the cage skeleton. In fact, see from the crystal structures, the H-OH-DABCO^2+^ is almost the largest cation that the cage skeleton constructed by NH_4_^+^-ClO_4_^−^ can hold. The potential hydrogen bonding interactions between H-OH-DABCO^2+^ and NH_4_^+^-ClO_4_^−^ skeleton during the heating conditions may also weaken the overall stability of the molecular perovskite.

**Figure 5:**
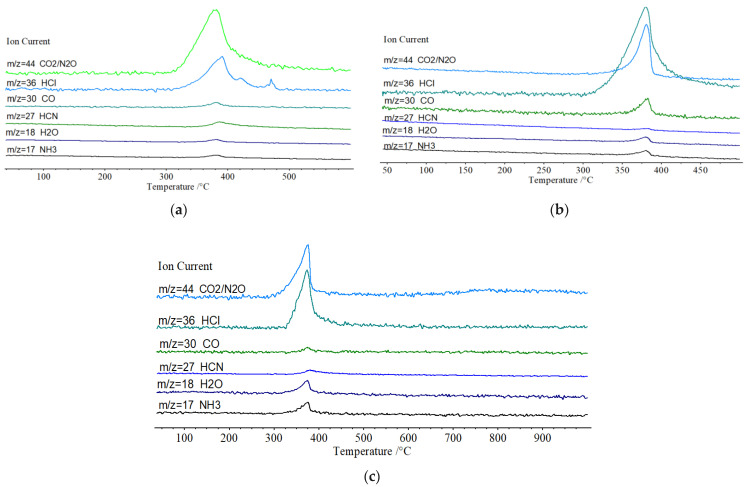
The mass spectra for (C_6_H_14_N_2_)[Na(ClO_4_)_3_] (**a**), (C_6_H_14_N_2_)[NH_4_(ClO_4_)_3_], (**b**) and (C_6_H_14_ON_2_)[NH_4_(ClO_4_)_3_] (**c**) at a heating rate of 10 °C/min.

Although in-situ FTIR spectroscopy method can provide rich information of the microscopic real-time changes of the condensed-phase during the thermolysis process, it cannot provide the information of microscopic real-time changes of the gaseous-phase products, which is also significant to clarify the thermal decomposition mechanism of energetic materials. For further investigations of the synergistic thermal decomposition mechanisms of the molecular perovskites, DSC-TG-FTIR-MS quadruple technology was further applied to perform the real-time and continuous analysis of their thermal decompositions. The anion (ClO_4_^−^) and cations (H-OH-DABCO^2+^ and H_2_DABCO^2+^) contained in the molecular perovskites represent the oxidant component and fuel component, respectively. With the increase in the heating temperatures, the Coulomb forces weakened and the cage skeletons collapsed, which led to the redox process between the ionic components, and the formations of gaseous-phase products [29,30]. Figure 5a–c showed the mass spectra of the gaseous-phase systems of (C_6_H_14_N_2_)[Na(ClO_4_)_3_], (C_6_H_14_N_2_)[NH_4_(ClO_4_)_3_], and (C_6_H_14_ON_2_)[NH_4_(ClO_4_)_3_] at a heating rate of 10 °C/min, respectively. The temperatures at which the gaseous decomposition products are detected are basically consistent with the experimental results obtained by DSC-TG and in situ FTIR methods, indicating same order of thermal stability of the three molecular perovskites in other experiments. Meanwhile, the major gaseous products from their decompositions include H_2_O (*m*/*z* = 18), HCN (*m*/*z* = 27), NH_3_ (*m*/*z* = 17), CO_2_ (*m*/*z* = 44), CO (*m*/*z* = 30), N_2_O (*m/z* = 44), HCl (*m*/*z* = 36). The similar decomposition products show similar decomposition mechanisms as follows: (1) the organic cations (H-OH-DABCO^2+^ and H_2_DABCO^2+^) was activated before the cage skeletons in which they are located under heating conditions and the hydrogen bonding interactions between the organic cations and ClO_4_^−^ gradually weaken the stabilities of the cage skeletons constructed by NH_4_^+^-ClO_4_^−^ and Na^+^-ClO_4_^−^; (2) after the interactions between the organic cations and ClO_4_^−^ are strong enough to destroy the cage skeletons, redox reactions are triggered in which the organic components (including the inorganic cation of NH_4_^+^) are oxidized, forming CO, CO_2_, H_2_O, HCN, and N_2_O. In contrast, the ClO_4_^−^ is reduced to HCl.

## 3. Methods

The thermal analysis includes DSC, DSC-TG, in situ FTIR, and DSC-TG-FTIR-MS quadruple technology experiments. Thermal analysis experiments were carried out with model TG-DSC STA 449C instrument (NETZSCH, Germany) and DSC Q200 instrument (TA, America). Operation conditions: sample mass, 0.5 mg; atmosphere, dynamic nitrogen; aluminum cell. IR spectra were recorded on a Nicolet 60SX FTIR spectrometer with HgCdTe detector. In situ FTIR spectroscopy studies were carried out with Nicolet 60 SXR FTIR spectrometer. Operation conditions: sample mass, 0.5 mg; heating rate, 10 °C/min; resolution, 4 cm^−1^; spectral acquisition rate, 17.8 file/min, 16 scans/file; temperature range, 20~450 °C. Cautions and experimental condition can be found in Supplementary Materials File S1.

## 4. Conclusions

In summary, the detailed thermal behaviors and decomposition mechanisms of the energetic molecular perovskites were investigated based on different experimental and calculation methods. The thermal stabilities of the molecular perovskites were determined by the applications of different organic cations (H-OH-DABCO^2+^ or H_2_DABCO^2+^) and cage skeletons constructed by NH_4_^+^-ClO_4_^−^ or Na^+^-ClO_4_^−^. Under heating conditions, the strength and thermal stability of the cage skeleton constructed by NH_4_^+^-ClO_4_^−^ are better than those of the cage skeleton constructed by Na^+^-ClO_4_^−^, and the thermal stability of the organic cation H_2_DABCO^2+^ is better than that of H-OH-DABCO^2+^ locked in the same cage skeleton. The major gaseous products from the decompositions molecular perovskites are similar, indicating similar decomposition mechanisms including cage skeleton strength weakening processes and redox reactions between the organic fuel components and inorganic oxidizer component of ClO_4_^−^. This work has improved the understanding of the decomposition processes and mechanisms of energetic molecular perovskites, and provide experimental basis of their further application in advanced explosives and propellants.

## Figures and Tables

**Table 1:** Kinetic parameters of the energetic molecular perovskites.

	Dynamic Analysis Model					
(C_6_H_14_N_2_) [Na(ClO_4_)_3_]	Kamal-Sourour autocatalytic model (KS)					
	Activation Energy (Ea_1_, kJ/mol)	LogA	React order n	Activation Energy (Ea_2_, kJ/mol)	Log(AutocatA)	Autocat Power m
	173.9	12.5	2.4	218.5	4.8	1.7
(C_6_H_14_N_2_) [NH_4_(ClO_4_)_3_]	nth order autocatalytic model (C_n_)					
	Activation Energy (Ea, kJ/mol)	LogA		React order n	Log(AutocatA)	
	207.5	13.8		0.6	0.5	
(C_6_H_14_ON_2_) [NH_4_(ClO_4_)_3_]	nth order model (F_n_)					
	Activation Energy (Ea, kJ/mol)	LogA			React order n	
	221.7	15.6			0.5	

Note: A: pre-exponential constant; Autocat: autocatalytic; Ea: activation energy; n: react order.

## Data Availability

All data generated or analyzed during this study are included in this published article.

## Supplementary Materials

The following are available online, File S1: Cautions! Experimental Condition.

## Sample Availability

Samples of the three energetic molecular perovskites are available from the authors.
