# Supplementary material for: Comparative Thermal Research on Energetic Molecular Perovskite Structures
Source: Molecules. 2022 Jan 26;27(3):805. doi: 10.3390/molecules27030805 (PMC8840576; doi:10.3390/molecules27030805)
Supplement: Supplementary file 1 [file molecules-27-00805-s001.zip › molecules-1544272-supplementary.pdf]

Comparative Thermal Research on Energetic Molecular Perovskite Structures

Jing Zhou [1,2], Junlin Zhang [1,*], Shaoli Chen [1], Fengqi Zhao [1], Lili Qiu [2,*], Zihui Meng [2], Li Ding [1], Bozhou Wang [1,*] and Qing Pan [1]

1 Xi'an Modern Chemistry Research Institute, Xi'an 710065, China; zhoujing19872006@163.com (J.Z.); asierchen@163.com (S.C.); zhaofqi@163.com (F.Z.); dingli403@sina.com (L.D.); IR204@tom.com (Q.P.)

2 School of Chemistry and Chemical Engineering, Beijing Institute of Technology, Beijing 102488, China; mengzh@bit.edu.cn

* Correspondence: junlin-111@163.com (J.Z.); qiulili@bit.edu.cn (L.Q.); wbz600@163.com (B.W.)

Cautions!

The described compounds are energetic materials with sensitivity to various stimuli. While we encountered no issues in the handling of these materials, proper protective measures should be used at all times.

Experimental Condition

All chemicals were obtained from commercial sources and used without further purification. The salts of $(C_6H_{14}ON_2)[NH_4(ClO_4)_3]$, $(C_6H_{14}N_2)[Na(ClO_4)_3]$, $(C_6H_{14}N_2)[NH_4(ClO_4)_3]$ were prepared and analyzed based on reported procedures. Their crystal data can be found in CCDC database. [1] (Reported by Dr. Xiaoming Chen and Dr. Weixiong Zhang group.)

The synthesis of $(C_6H_{14}ON_2)[NH_4(ClO_4)_3]$ salt was carried out based on the literature method[2]: NH_4ClO_4 (2 mmol) and 70% $HClO_4$ (0.7 mL) solution were added to H_2O (5 mL), then an aqueous solution (2 mL) of DABCO-O (2 mmol) was added into the mixture slowly. White solid was then collected by filtration.

The synthesis of $(C_6H_{14}N_2)[Na(ClO_4)_3]$, $(C_6H_{14}N_2)[NH_4(ClO_4)_3]$ salts were carried out based on the literature method[3]: Sodium/ammonium perchlorate (1 mmol) was added into an aqueous solution (20 mL) of triethylenediamine (1 mmol), then 70% $HClO_4$ solution was added into the mixture. $(C_6H_{14}N_2)[Na(ClO_4)_3]$, $(C_6H_{14}N_2)[NH_4(ClO_4)_3]$ salts were collected by filtration.

[1] CCDC numbers: 1956808, 1528107 and 1528108.

[2] Shang, Y., Huang, RK., Chen, SL. *et al. Cryst. Growth Des.* 20, 1891–1897 (2020). https://doi.org/10.1021/acs.cgd.9b01592

[3] Chen, SL., Yang, ZR., Wang, BJ. *et al.* Molecular perovskite high-energetic materials. *Sci. China Mater.* **61,** 1123–1128 (2018).

https://doi.org/10.1007/s40843-017-9219-9

The thermal analysis includes DSC, DSC-TG, in-suit FTIR and DSC-TG-FTIR-MS quadruple technology experiments. Thermal analysis experiments were carried out with model TG-DSC STA 449C instrument (NETZSCH, Germany) and DSC Q200 instrument (TA, America). Operation conditions: sample mass, 0.5 mg; atmosphere, dynamic nitrogen; aluminum cell. IR spectra were recorded on a Nicolet 60SX FTIR spectrometer with HgCdTe detector. In-situ FTIR spectroscopy studies were carried out with Nicolet 60 SXR FTIR spectrometer. Operation conditions: sample mass, 0.5 mg; heating rate, 10 °C·min^{-1}; resolution, 4 cm^{-1}; spectral acquisition rate, 17.8 file·min^{-1}, 16 scans·file^{-1}; temperature range, 20~450 ^{o}C.
